# Quarternization of 3-azido-1-propyne oligomers obtained by copper(I)-catalyzed azide–alkyne cycloaddition polymerization

**DOI:** 10.3762/bjoc.11.116

**Published:** 2015-06-18

**Authors:** Shun Nakano, Akihito Hashidzume, Takahiro Sato

**Affiliations:** 1 Department of Macromolecular Science, Graduate School of Science, Osaka University, 1-1 Machikaneyama-cho, Toyonaka, Osaka 560-0043, Japan

**Keywords:** 3-azido-1-propyne oligomer, CuAAC polymerization, hydrodynamic radius, methyl iodide, pulse-field-gradient spin-echo NMR, quarternization

## Abstract

3-Azido-1-propyne oligomer (oligoAP) samples, prepared by copper(I)-catalyzed azide–alkyne cycloaddition (CuAAC) polymerization, were quarternized quantitatively with methyl iodide in sulfolane at 60 °C to obtain soluble oligomers. The conformation of the quarternized oligoAP in dilute DMSO-*d*_6_ solution was examined by pulse-field-gradient spin-echo NMR based on the touched bead model.

## Introduction

The copper(I)-catalyzed azide–alkyne cycloaddition (CuAAC) efficiently yields 1,4-disubstituted-1,2,3-triazole from rather stable azides and alkynes with a copper(I) catalyst under mild conditions even in the presence of various functional groups [1–3]. CuAAC is thus the most important reaction in “click chemistry” [4–7]. A number of studies have been published on CuAAC, which is applied in a wide range of fields from bio-related chemistry [8–12] to materials science [13–29], as well as polymer synthesis [30–36]. Most of these studies deal with the 1,2,3-triazole moiety just as a linker. However, since 1,2,3-triazole possesses a large dipole moment and aromaticity, 1,2,3-triazole itself may be a functional group. Polymers composed of dense 1,2,3-triazole moieties on the backbone are thus promising as functional materials.

**Scheme 1:**
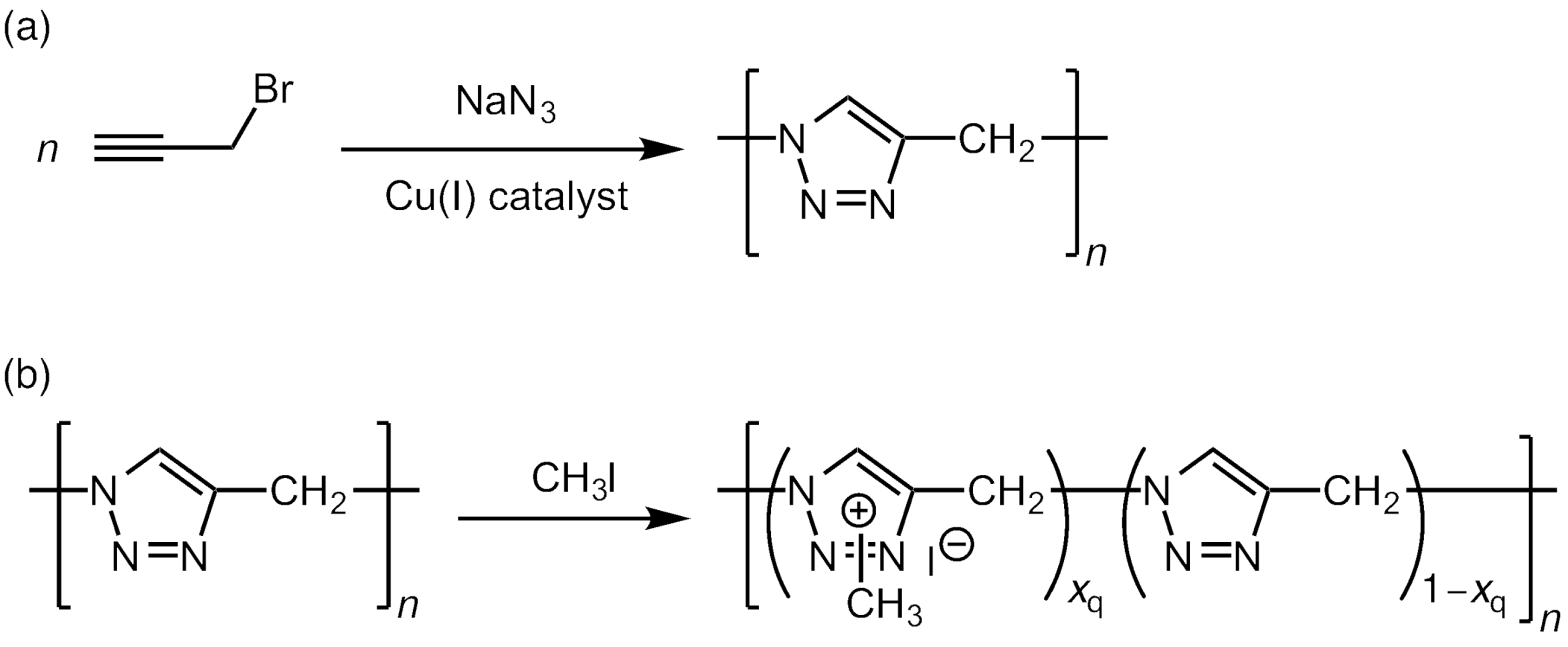
CuAAC polymerization of 3-azido-1-propyne (AP) (a) and quarternization of 3-azido-1-propyne oligomer (oligoAP) with methyl iodide (b).

Recently, we have investigated the CuAAC polymerization of 3-azido-1-propyne (AP) using 3-bromo-1-propyne as a monomer precursor, and obtained its oligomer, whose backbone is composed of 1,2,3-triazole and methylene moieties (Scheme 1a) [37]. The oligomer obtained was crystalline and adsorbed strongly copper ions. Since the oligomer was soluble only in strong acids and insoluble in water and organic solvents, it was not possible to characterize the oligomer in solution. The observation that the AP oligomer is well soluble in strong acids suggests that protonation of the 1,2,3-triazole moieties improves its solubility. Thus we were motivated to study quarternization of the AP oligomer because it is known that 1,2,3-triazole is quarternized with alkyl halides or others [38–39]. Recently, it has been reported that quarternized 1,2,3-triazole derivatives act as ionic liquids [40–41], and polymeric ionic liquids have been also prepared from polymers possessing 1,2,3-triazole moieties [42–44]. Therefore the quarternized AP oligomer may be applied as a polymeric ionic liquid or a precursor of polymeric ionic liquids. In this study, we investigate quarternization of an AP oligomer with methyl iodide to obtain a soluble oligomer (Scheme 1b) and characterize the quarternized oligomer in dilute solutions.

## Results and Discussion

Table 1 lists the conditions and the results of quarternization of the oligomer of 3-azido-1-propyne (oligoAP) with a degree of polymerization *n* = 14. As can be seen in Table 1, oligoAP was quarternized almost quantitatively with a large excess of methyl iodide in sulfolane at 60 °C for 24 h (Table 1, run 1). The degree of quarternization (*x*_q_) was determined to be ca. 0.94 by ^1^H NMR as described in the later subsection. The degree of quarternization *x*_q_ can be controlled by altering the amount of methyl iodide used (see runs 2–4 in Table 1).

**Table 1:** Conditions and results of quarternization of oligoAP with methyl iodide.^a^

run	the amount of monomer unit in the oligoAP used / mmol	the amount of methyl iodide used / mmol	time / h	yield / %	*x*_q_^b^

1	2.6	80	24	54	0.94
2	1.9	0.76	16	58	0.30
3	1.9	0.38	16	68	0.20
4	2.0	0.19	16	77	0.11

^a^Quarternization was carried out using oligoAP with a degree of polymerization *n* = 14 in sulfolane at 60 °C. ^b^The degree of quarternization (see Scheme 1b).

**Figure 1:**
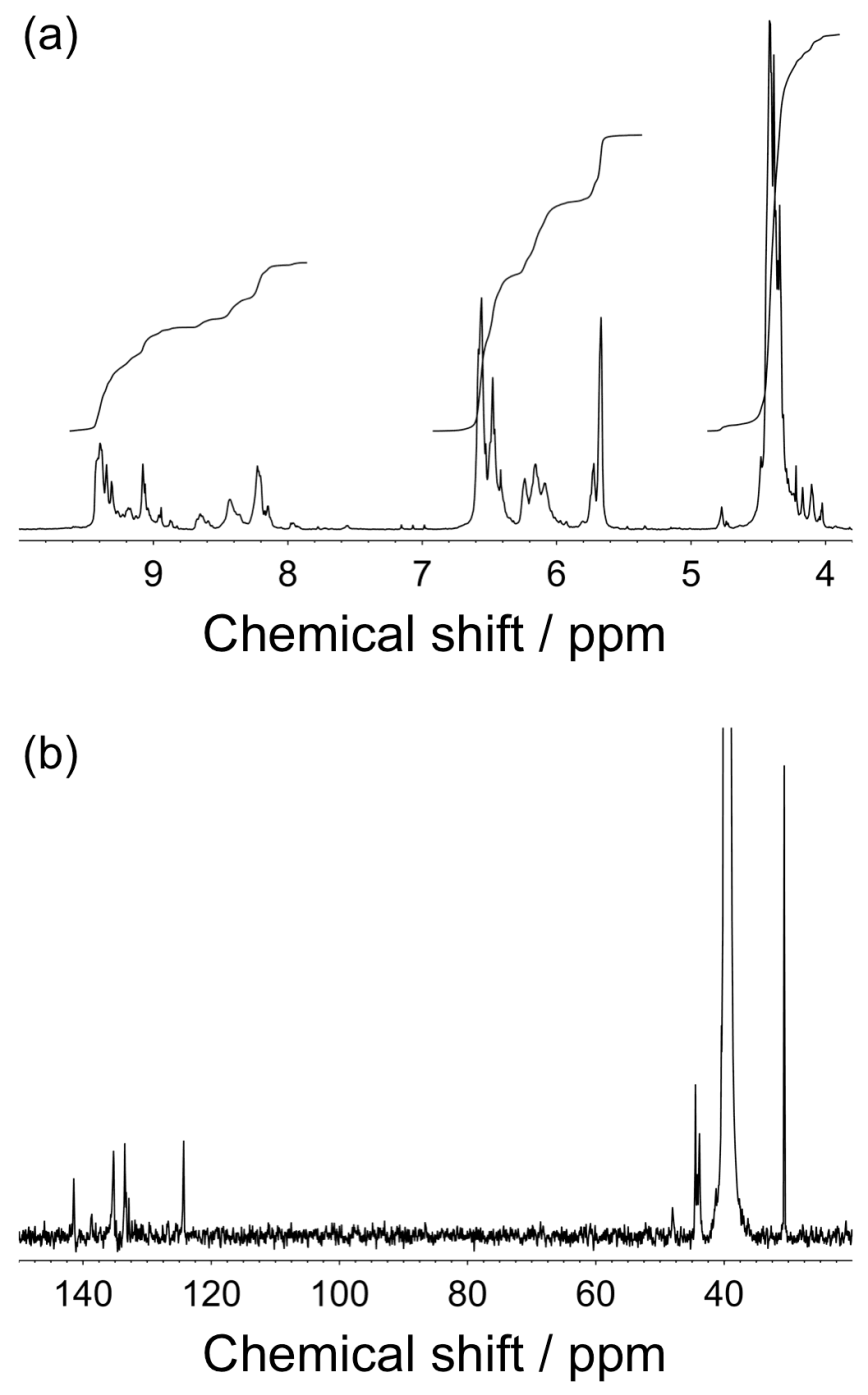
600 MHz ^1^H (a) and 125 MHz ^13^C NMR spectra for oligoAPMe of *n* = 14 and *x*_q_ = 0.94 measured in DMSO-*d*_6_ at 30 °C (b).

Figure 1 displays a typical example of ^1^H and ^13^C NMR spectra measured in dimethyl sulfoxide-*d*_6_ (DMSO-*d*_6_) for the oligomer quarternized (oligoAPMe, run 1 in Table 1). As can be seen in Figure 1a, the ^1^H NMR spectrum exhibits signals ascribable to the methine proton in 1,2,3-triazole and methylene protons in the polymer backbone in the region of 9.4–7.9 and 6.6–5.6 ppm, respectively. The spectrum also contains signals assignable to the methyl groups introduced in the range of 4.6–4.0 ppm. In the ^13^C spectrum (Figure 1b), there are signals due to the methylene and methyl carbons at ca. 44 and 30.6 ppm, respectively. In the aromatic region of the ^13^C NMR spectrum, the signals at 141.4, 135.2, 133.5, and 124.3 ppm are ascribable to the carbons in the 1,2,3-triazole ring. This observation indicates that the methylation occurred on both the 2- and 3-positions in 1,2,3-triazole under the present conditions, although the quarternization of 1,2,3-triazole usually occurs on the 3-position [40,43–44]. From the ratio of the integrals of signals due to the methylene and methyl protons, *x*_q_ was calculated as listed in Table 1. The ^1^H NMR spectrum confirms that oligoAP was successfully quarternized and the oligoAP quarternized with methyl iodide was soluble in DMSO-*d*_6_.

The C, H, and N contents of the oligoAPMe of *x*_q_ = 0.94 (C, 22.12; H, 2.74; N, 19.68) were in agreement with those calculated for oligoAP quarternized quantitatively (C, 22.30; H, 2.97; N, 19.94). The elemental analysis data indicated that the oligoAPMe did not contain inorganic compounds although it was not possible to remove completely inorganic compounds from oligoAP even after washing repeatedly with a saturated aqueous solution of ethylenediaminetetraacetic acid (EDTA) and 5% nitric acid [37]. These observations indicate that quarternization weakens the interaction with metal ions.

While oligoAP was insoluble in all the solvents examined, including, DMSO, water, methanol, acetone, THF, and toluene [37], the oligoAPMe of *x*_q_ = 0.94 was soluble in DMSO and slightly soluble in water and methanol. The solubility of oligoAPMe in DMSO increased with *x*_q_. The oligoAPMe of *x*_q_ = 0.94 was insoluble in less polar solvents, acetone, THF, and toluene.

**Figure 2:**
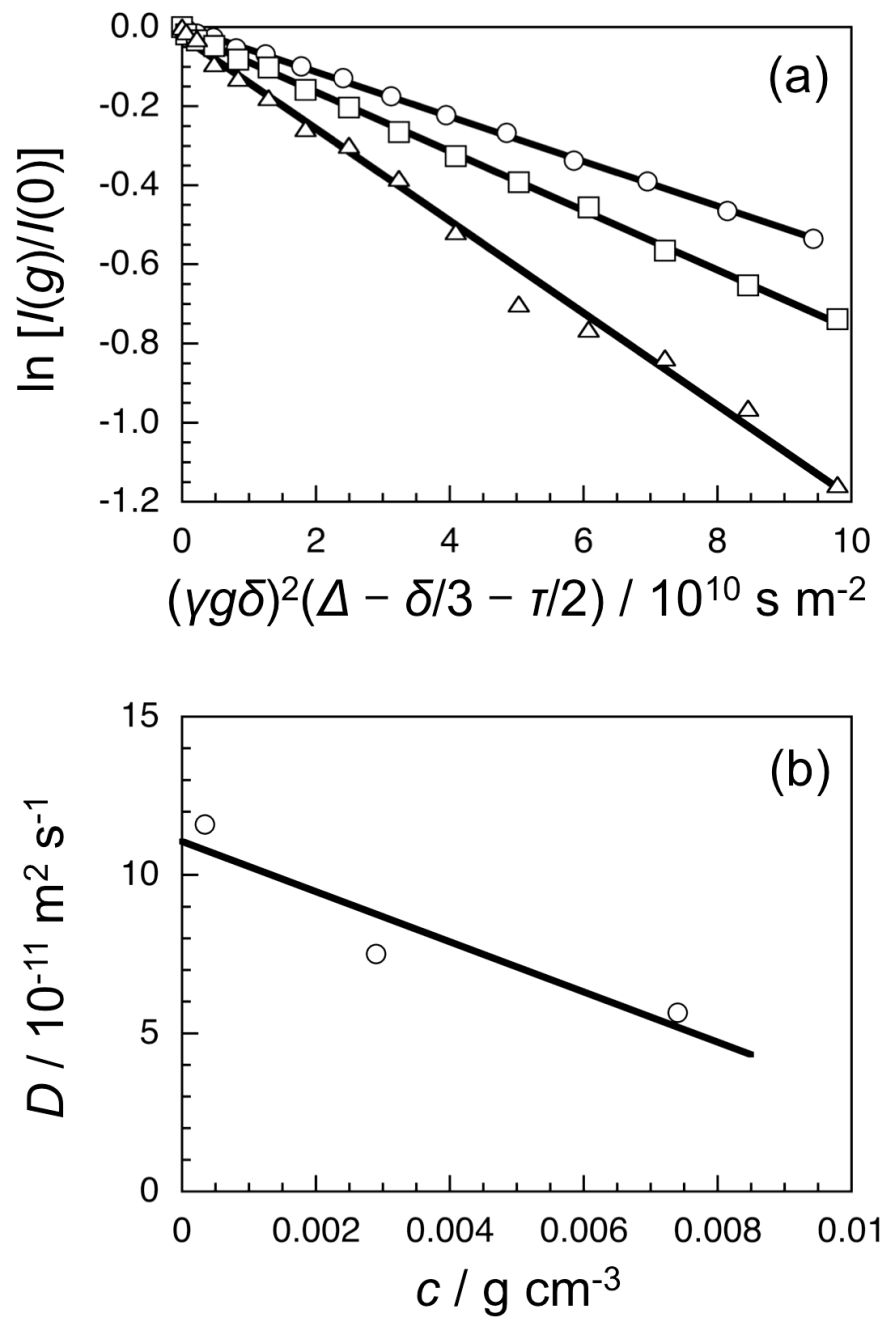
PGSE NMR spectroscopic data for dilute DMSO-*d*_6_ solutions of oligoAPMe with *n* = 11 and *x*_q_ = 0.96 of three different concentrations (0.00034 (triangle), 0.0029 (square), and 0.0074 g cm^–3^ (circle)) at 30 °C, where ln[*I*(*g*)/*I*(0)] is the logarithm of the ratio of the observed spin echo intensities with and without field gradients, *γ* the gyromagnetic ratio, and *τ* the 90°–180° pulse distance (a), and the concentration dependency of apparent self-diffusion coefficient *D* for the oligoAPMe in DMSO-*d*_6_ at 30 °C (b).

The conformation of quarternized oligoAP in DMSO-*d*_6_ was examined by pulse-field-gradient spin-echo (PGSE) NMR (see Supporting Information File 1). An oligoAPMe sample of *n* = 11 and *x*_q_ = 0.96 was used for the experiment. The intensities of echo signals at ca. 8.9 ppm were evaluated as a function of the intensity of the pulse-filed-gradient for dilute DMSO-*d*_6_ solutions of the oligoAPMe of three different concentrations, as can be seen in Figure 2a. Using the Sterjkal equation, the self-diffusion coefficients (*D*) were determined from the slopes of the straight lines in Figure 2a, and extrapolated to the zero concentration to obtain the limiting diffusion coefficient (*D*_0_) (Figure 2b). It should be noted here that the extrapolation to the zero concentration is rather normal, indicating that the iodide counterions of oligoAPMe are not considerably dissociated in DMSO-*d*_6_. From the *D*_0_ value, the hydrodynamic radius (*R*_H_) was estimated to be 0.95 nm using the Einstein–Stokes equation.

**Figure 3:**
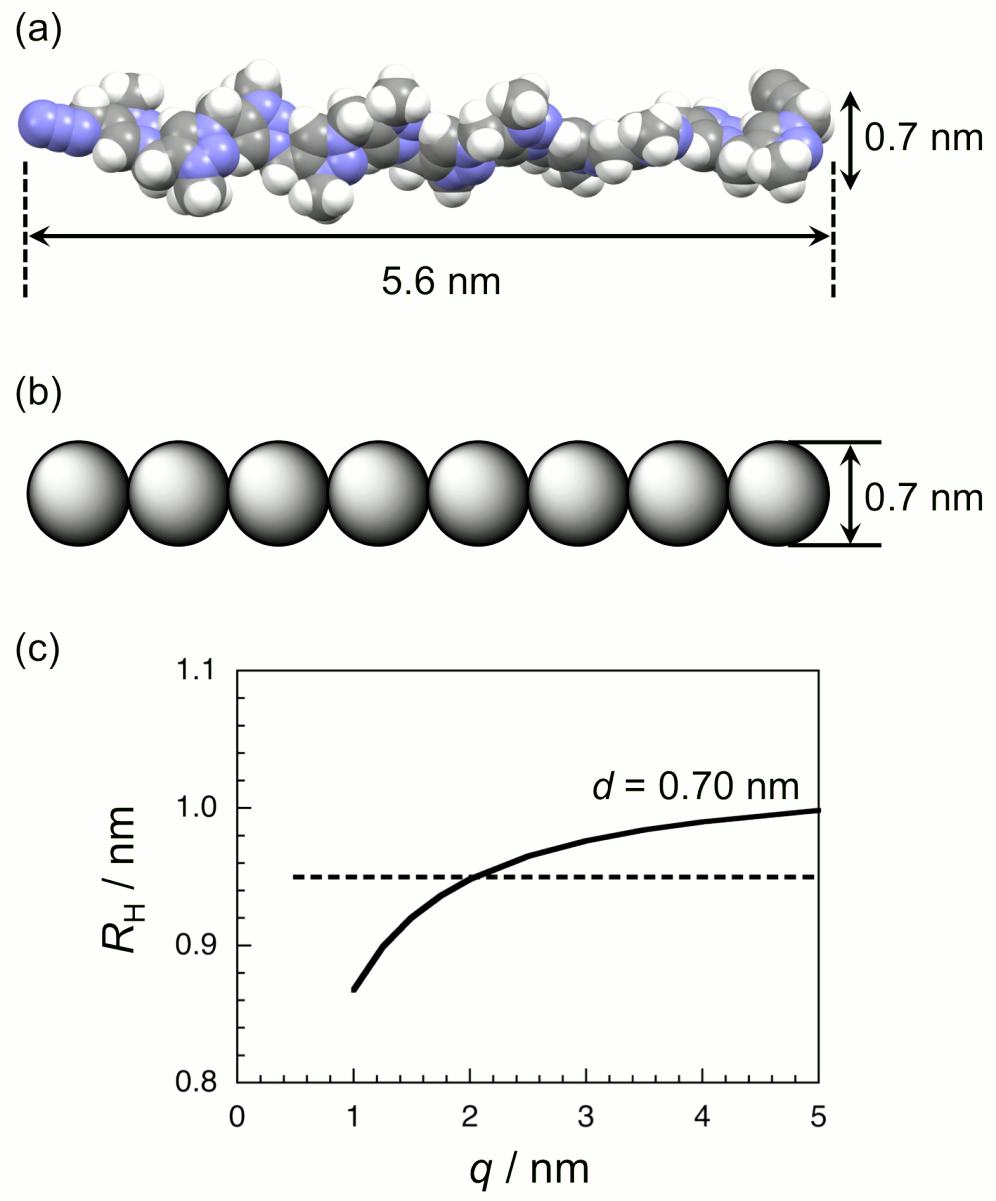
A typical example of the three-dimensional structure of oligoAPMe of *n* = 11 built with ChemBio3D software (version 13.0) (a), a rodlike touched bead model with a bead diameter (*d*) of 0.7 nm for the oligoAPMe (b), and the hydrodynamic radius *R*_H_ as a function of the persistence length *q* for the diameter *d* of 0.70 nm based on the touched bead model (c). The broken line presents the experimental *R*_H_ (= 0.95 nm).

Figure 3a illustrates an extended conformation of oligoAPMe of *n* = 11 built with a ChemBio3D software (version 13.0). Here all the methyl groups are attached onto the 3-position for simplicity. From this space-filling model, the contour length of the oligoAPMe was estimated to be 5.6 nm. When this oligomer model is viewed as a rodlike touched bead model with a bead diameter (*d*) of 0.7 nm (Figure 3b), *R*_H_ is calculated to be 1.03 nm [45]. This value is larger than the experimental *R*_H_ (= 0.95 nm) for oligoAPMe. If *d* of the rodlike touched bead model is chosen to be 0.55 nm, the theoretical *R*_H_ agrees with the experimental one. However, 0.55 nm seems to be too thin as seen from Figure 3a. If the oligomer is viewed as a touched bead wormlike chain model with *d* = 0.7 nm and the persistence length (*q*) = 2 nm, the theoretical *R*_H_ [45–46] is in agreement with the experimental one (cf. Figure 3c). A more precise conformational analysis of oligoAP quarternized should be studied in future.

## Conclusion

Quarternization of oligoAP was investigated using methyl iodide to obtain soluble oligomers; oligoAP was quarternized quantitatively with a large excess of methyl iodide in sulfolane at 60 °C. The oligoAPMe of *x*_q_ = 0.94 was soluble in DMSO, indicating that quarternization makes the oligoAP soluble. The ^1^H and ^13^C NMR spectra for the oligoAPMe indicated that the methylation occurred on both the 2- and 3-positions in 1,2,3-triazole under the present conditions. The hydrodynamic radius *R*_H_ of oligoAPMe of *n* = 11 and *x*_q_ = 0.96 in DMSO-*d*_6_ dilute solutions was determined to be 0.95 nm by PGSE NMR to study the conformation of the quarternized oligomer. The conformation of oligoAPMe was further discussed based on the touched bead model. Since the oligoAPMe obtained in this study was solid and the iodide counterions were not significantly dissociated in DMSO, it is difficult to apply the oligoAPMe as a polymeric ionic liquid. However, if appropriate quarternization reagents and counterions are employed, a new type of polymeric ionic liquids may be provided.

## Experimental

IR spectra were recorded on a JASCO FT/IR-8300 spectrometer equipped with a JASCO ATR PRO410-S cell. ^1^H and ^13^C NMR spectra were recorded on an Agilent 600 NMR spectrometer at 30 °C using DMSO-*d*_6_ as a solvent. Chemical shift values were referenced to the solvent values (2.50 and 39.5 ppm for ^1^H and ^13^C, respectively). PGSE NMR spectra were also recorded on an Agilent 600 NMR spectrometer at 30 °C using DMSO-*d*_6_ as a solvent. A bipolar pulse pair stimulated echo (BPPSTE) sequence was applied [47–49]. The strength of pulsed gradients (*g*) was increased from 6.36 × 10^−1^ to 43.1 gauss cm^–1^. The time separation of pulsed field gradients (Δ) and their duration (δ) were 0.10 and 2.0 × 10^−3^ s, respectively. The sample was not spun and the airflow was disconnected. The shape of the gradient pulse was rectangular, and its strength varied automatically during the course of the experiments.

3-Bromo-1-propyne (BrP), sodium azide (NaN_3_), *N*,*N*-dimethylformamide (DMF), copper sulfate pentahydrate (CuSO_4_·5H_2_O), (+)-sodium L-ascorbate (NaAsc), and methyl iodide were purchased from Wako Pure Chemical Industry, Ltd. DMF, used for polymerization, was purified by distillation under reduced pressure over calcium hydride just prior to use. Water was purified by a Millipore Milli-Q system. Other reagents were used without further purification.

Samples of oligoAP were prepared by CuAAC polymerization using BrP and CuSO_4_·5H_2_O/NaAsc as a monomer precursor and a catalyst, respectively, in 70–90 % yield, as described previously [37]. The oligoAP samples were purified by washing repeatedly with water (2 × 60 mL), a saturated aqueous solution of EDTA (3 × 60 mL), and then water (2 × 60 mL). The degrees of polymerization (*n*) were estimated to be ca. 11 and 14 by NMR and IR [50].

A typical procedure of quarternization of oligoAP is described below. The oligoAP sample of *n* = 14 (0.21 g) was placed in a 100 mL flask under an argon atmosphere. A solution of methyl iodide (5 mL, 80 mmol) in sulfolane (6 mL) was added into the flask. The reaction mixture was warmed with an oil bath thermostated at 60 °C with stirring. After 24 h, the reaction mixture was poured into THF (100 mL) for termination of the reaction. The precipitate formed was washed with THF (2 × 50 mL) and diethyl ether (2 × 60 mL). After drying under reduced pressure, the polymer obtained was recovered as yellow powder: yield 0.32 g, 54%; mp >300 °C dec; ^1^H NMR (DMSO-*d*_6_, 500 MHz) δ 4.0–4.6 (methyl), 5.6–6.6 (methylene), 7.9–9.4 (triazole methine); ^13^C NMR (DMSO-*d*_6_, 125 MHz) δ 141.4 (triazole), 135.2 (triazole), 133.5(triazole), 124.3 (triazole), 44.5 (methylene), 44.2 (methylene), 43.9 (methylene), 30.6 (methyl); calcd for C_59_H_87_N_45_I_14_: C, 22.30; H, 2.97; N, 19.94 %; found: C, 22.12; H, 2.74; N, 19.68 %.

## Supporting Information

File 1 Estimation of the apparent self-diffusion coefficient for the oligomer quarternized.
