# Supplementary material for: Quarternization of 3-azido-1-propyne oligomers obtained by copper(I)-catalyzed azide–alkyne cycloaddition polymerization
Source: Beilstein J Org Chem. 2015 Jun 18;11:1037–42. doi: 10.3762/bjoc.11.116 (PMC4505306; doi:10.3762/bjoc.11.116)
Supplement: File 1 — Estimation of the apparent self-diffusion coefficient for the oligomer quarternized. [file Beilstein_J_Org_Chem-11-1037-s001.pdf]

Supporting Information

for

# Quarternization of 3-azido-1-propyne oligomers obtained by copper(I)-catalyzed azide–alkyne cycloaddition polymerization

Shun Nakano, Akihito Hashidzume*, and Takahiro Sato

Address: Department of Macromolecular Science, Graduate School of Science, Osaka University, 1-1 Machikaneyama-cho, Toyonaka, Osaka 560-0043, Japan

Email: Akihito Hashidzume - akihito@chem.sci.osaka-u.ac.jp

*Corresponding author

## Estimation of the apparent self-diffusion coefficient for the oligomer quarternized

Figure 2a shows a typical example of the logarithm of the ratio of the observed spin echo intensities with and without field gradients ($\ln[I(g)/I(0)]$) as a function of $\gamma^2 g^2 \delta^2(\Delta - \delta/3 - \tau/2)$ for oligoAP**Me**, where $\gamma$ is the gyromagnetic ratio, $g$ the gradient strength, $\Delta$ the delay between the midpoints of the gradients, $\delta$ the gradient length, and $\tau$ the 90°–180° pulse distance. From the slopes of the straight lines and the Sterjkal equation [1-3]

$$\ln[I(g)/I(0)] = -\gamma^2 g^2 \delta^2(\Delta - \delta/3 - \tau/2)D \qquad \text{(S1)}$$

the apparent self-diffusion coefficients ($D$) were determined.

# References


1. Stejskal, E. O.; Tanner, J. E. *J. Chem. Phys.* **1965**, *42*, 288-292.

2. Tanner, J. E.; Stejskal, E. O. *J. Chem. Phys.* **1968**, *49*, 1768-1777.

3. Stilbs, P. *Prog. Nucl. Magn. Reson. Spectrosc.* **1987**, *19*, 1-45.
